# Regulating ehrlich and demethiolation pathways for alcohols production by the expression of ubiquitin-protein ligase gene *HUWE1*

**DOI:** 10.1038/srep20828

**Published:** 2016-02-10

**Authors:** Quan Zhang, Kai-Zhi Jia, Shi-Tao Xia, Yang-Hua Xu, Rui-Sang Liu, Hong-Mei Li, Ya-Jie Tang

**Affiliations:** 1 Key Laboratory of Fermentation Engineering (Ministry of Education), Hubei Provincial Cooperative Innovation Center of Industrial Fermentation, Hubei Key Laboratory of Industrial Microbiology, Hubei University of Technology, Wuhan 430068 China

## Abstract

Ehrlich and demethiolation pathways as two competing branches converted amino acid into alcohols. Controlling both pathways offers considerable potential for industrial applications including alcohols overproduction, flavor-quality control and developing new flavors. While how to regulate ehrlich and demethiolation pathways is still not applicable. Taking the conversion of methionine into methionol and methanethiol for example, we constructed two suppression subtractive cDNA libraries of *Clonostachys rosea* by using suppression subtractive hybridization (SSH) technology for screening regulators controlling the conversion. E3 ubiquitin-protein ligase gene *HUWE1* screened from forward SSH library was validated to be related with the biosynthesis of end products. Overexpressing *HUWE1* in *C. rosea* and *S. cerevisiae* significantly increased the biosynthesis of methanethiol and its derivatives in demethiolation pathway, while suppressed the biosynthesis of methional and methionol in ehrlich pathway. These results attained the directional regulation of both pathways by overexpressing *HUWE1*. Thus, *HUWE1* has potential to be a key target for controlling and enhancing alcohols production by metabolic engineering.

Ehrlich and demethiolation pathways, two major branches for catabolizing amino acid, is received much attention for both pathways significantly participated to the production of fuel and valuable flavor alcohols, the quality and uniqueness of many foodstuffs^1^,^2^,^3^,^4^,^5^. L-phenylalanine was catabolized into 2-phenylethanol which is widely used in perfumery and cosmetics for its rose-like odor^6^, valine and leucine were converted into butanol, isobutanol and 3-methyl-1-butanol as fuels via ehrlich pathway^3^,^5^. Additionally, catalyzing methionine (Met) into methional and methionol via ehrlich pathway, methanethiol (MTL) and its derivatives dimethyl sulfide (DMS), dimethyl disulfide (DMDS) and dimethyl trisulfide (DMTS) via demethiolation pathway is of prime importance in the overall flavor formation of fermented food and make a significant contribution to their typical flavors^4^. In order to improve the production of alcohols or control the quality of fermented food, it is necessary to cost-effectively engineer fermentation strains to fine-tune the ehrlich and demethiolation pathways.

The ubiquitin-proteasome system (UPS), regulating cellular homeostasis, was involved in numerous cellular processes including cell cycle progression, gene transcription, protein quality control, and signal transduction^7^,^8^. Ubiquitination is achieved through the action of three enzyme classes: E1-ubiquitin-activating enzymes, E2-ubiquitin-conjugating enzymes, and E3-ubiquitin ligases^8^. Of which E3-ubiquitin ligases played critical role during post-translational modifications for they polyubiquitinate their substrates with lys48-linked chains of ubiquitin and target the substrates for destruction by the proteasome^9^. These substrates included transcriptional factors and catabolic enzymes^9^. Up to now, E3-ubiquitin ligases were found to regulate numerous cellular pathways closely related with carbon and lipid catabolites, stress adaptation^10^,^11^,^12^,^13^. Therefore, ubiquitination constituents, especially E3-ubiquitin ligases, are potential targets for regulating the complex metabolic switches.

Ehrlich pathway genes including aminotransferase genes *ARO8*, *BAT* and α-ketoacid decarboxylase gene *PDC* and demethiolation pathway gene *STR3* are highly conserved in eukaryotes. Their coordinated expression at transcriptional level controlled the degradation of amino acids^14^,^15^. The regulators performing key regulation tasks during the coordinated expression of pathway genes have potential to be targets for overproduction of metabolites by metabolic engineering^16^,^17^. Up to now, only one regulator ARO80 was found to activate the expression of ehrlich pathway genes encoding aminotransferases and α-ketoacid decarboxylases in response to aromatic amino acids^18^,^19^. Demethiolation pathway as competing branch directly impacted the biosynthesis of ehrlich pathway metabolites. Thus how to regulate the two pathways by metabolic engineering is closely related with controlling the metabolites compositions and concentrations which decided the alcohols production and food quality.

In this study, *Clonostachys rosea* tang 19 was selected for its ability to degrade Met into methionol and MTL via ehrlich and demethiolation pathways, respectively. To achieve rational regulation of the two pathways, we constructed a suppression subtractive cDNA library and screened differentially expressed genes especially regulator genes associated with the biosynthesis of end products. The effects of these regulators on the production of ehrlich and demethiolation pathway metabolites were analyzed in *Clonostachys rosea* and *Saccharomyces cerevisiae*. Our findings support the rational regulation of ehrlich and demethiolation pathways for enhancing alcohols production, the quality optimization and fermentation control.

## Results

### Differentially expressed genes associated with the conversion of Met into methionol and MTL

To enrich the differentially expressed genes associated with alcohols production, suppression subtractive hybridization PCR was adopted in this study. The optimal PCR cycle numbers were 17 for “Driver” cDNA and 14 for “Tester” cDNA. The PCR products of SMARTer cDNA were mainly distributed between 0.7 and 2.0 kb (Fig. S1A–D). Subsequently, subtractive cDNA libraries were constructed to isolate the differentially expressed fragments triggered by Met addition. A total of 2405 clones, ranging from 100 to 1500 bp for the two libraries, were obtained. The forward and reverse libraries containing 1272 clones and 1133 clones were constructed by using suppression subtractive hybridization (SSH) technology, respectively.

All PCR products ranged from 100 to 1500 bp were arrayed on 10 nylon membranes in duplicate and screened by cDNA array dot blotting. Apparent differences in hybridization signal intensities were observed when comparing the membrane probed with the forward probe to the membrane probed with the reverse subtracted probe (Fig. S2A–D), which indicated that SSH procedures for screening differentially expressed sequences were succeeded. 100 positive clones from forward and reverse libraries were sequenced and analyzed, respectively.

### Bioinformatics based functional inference of differentially expressed genes

**Figure 1:**
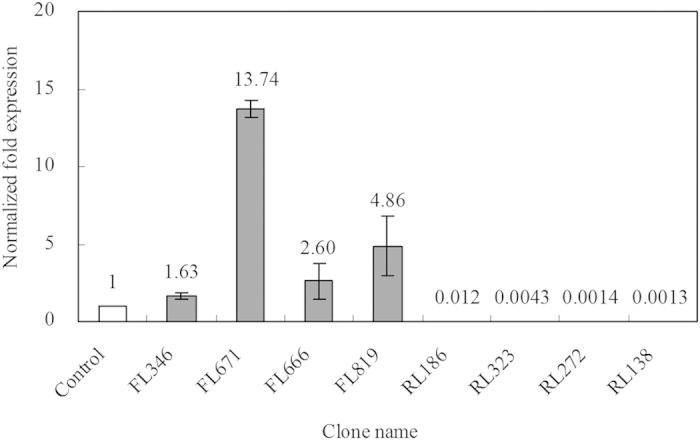
Quantitative real-time PCR analysis of differently expressed genes. Normalized fold expression values for aminotransferase genes *ARO8-2* and *BAT*, decarboxylase gene *PDC* and demethiolase gene *STR3* were relative to the control without Met addition. The error type was standard deviation.

The potential functions of expressed sequence tags from the libraries were inferred based on homology by using the basic local alignment search tool (BLASTX). 91 and 96 unigenes were obtained from the forward and reverse libraries (Tables 1 and 2). To validate the efficacy for screening differently expressed genes, 4 genes were selected from the forward or reverse libraries and their expression at transcriptional level was analyzed by using real-time PCR, respectively. After Met addition, the expression of FL346 encoding E3 ubiquitin-protein ligase HUWE1, FL671 encoding aromatic amino acid aminotransferase (*ARO8-2*), FL666 encoding pyruvate decarboxylase (*PDC*) and FL819 encoding 3-isopropylmalate dehydrogenase was increased by 1.63, 13.74, 2.60 and 4.86 folds, while the expression of RL186 encoding amino-acid permease inda1, RL323 encoding aminotransferase class I and II, RL272 encoding 5-aminolevulinate synthase and RL138 encoding polyketide synthase were decreased by 83.33, 232.56, 714.29 and 769.23 folds, respectively (Fig. 1). The transcriptional expression pattern of these genes met with the results obtained from SSH, which indicated that SSH library had been successfully constructed.

**Figure 2:**
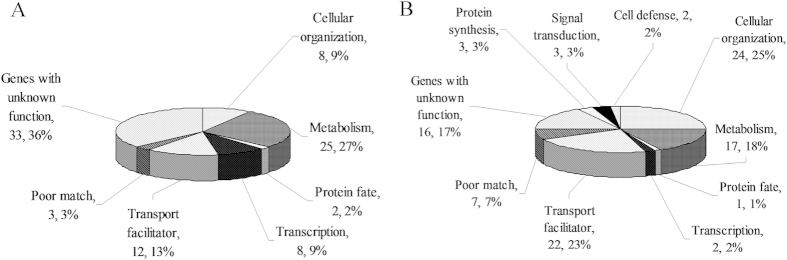
Functional categories analysis of cDNA sequences represented in (**A**) the forward SSH-cDNA library and (**B**) the reverse SSH-cDNA library. Functional categories are based on MIPS classification.

Gene ontology analysis categorized the gene set into 7 and 10 functional groups (Fig. 2A,B, Tables 1 and 2), respectively. Among these, cell metabolism, transcription and protein fate were three categories closely related with the conversion of Met into methionol and MTL. In cell metabolism, *ARO8-2* encoding aromatic amino acid aminotransferase and *PDC* encoding α-ketoacid decarboxylase were selected from forward library which may contribute to the biosynthesis of methionol via ehrlich pathway. Regulators functioned during the transcription of synthase genes which is key step for controlling methionol and MTL production (Tables 1 and 2). Transcription factor analysis revealed that 5 transcription factors including C6 (Cys6)^19^, F-box (mediating protein interaction, involved in cell cycle and signal transduction)^20^, PWWP (mediating nucleus protein interaction)^21^, Tar1p (related with mitochondrial gene expression and mtDNA stability)^22^ and Hms1p (repressing citrate biosynthesis)^23^ (Tables 1 and 2) were associated with the biosynthesis of methionol and MTL. Ubiquitin 3 binding protein But2, ubiquitin conjugating E2 and E3 ubiquitin-protein ligase HUWE1 were only three posttranscriptional regulators selected from protein fate group. Relative to ubiquitin 3 binding protein But2 and ubiquitin conjugating E2, E3 ubiquitin-protein ligase HUWE1 directly controlled ubiquitination process (Tables 1 and 2). E3 ubiquitin-protein ligases regulated the biosynthesis of metabolites by polyubiquitating proteins implicated in transcription, protein folding and degradation^24^. Thus they are ideal targets for large scale alteration of cellular metabolism by metabolic engineeing. The production of methionol and MTL is resulted from the coordinated transcriptional expression of synthase genes and may be controlled by a hierarchical transcriptional regulatory network^15^. In view to huge potential regulatory capability of HUWE1, HUWE1 is adopted to rationally regulate ehrlich and demethiolation pathways.

### Overexpression of *HUWE1* impacted the biosynthesis of pathway metabolites in *C. rosea*

*HUWE1* screened from protein fate group of forward SSH library, may contribute to the production of end products. Thus, we inferred that overexpression of *HUWE1* may further enhance the production of methionol and MTL. To overexpress *HUWE1* in *C. Rosea*, a 4140 bp sequence located in the upstream genomic region of *HUWE1* was cloned by SEFA PCR and then the full-length ORF encoding a 988 amino acid polypeptide was directly integrated into the genome of *C. Rosea*. Transcriptional analysis indicated that *HUWE1* was overexpressed indeed in overexpression strains U1 and U2 (Fig. S3A,B). E3 ubiquitin-protein ligase HUWE1 was characterized with HECT (homologous to E6-associated protein C-terminus) which has a direct role in catalysis during ubiquitination. To further identify that the two pathways are regulated by *HUWE1*, the sequence encoding catalysis domain HECT was deleted from *HUWE1* and then the residue fragment was overexpressed in *C. Rosea.*

**Figure 3:**
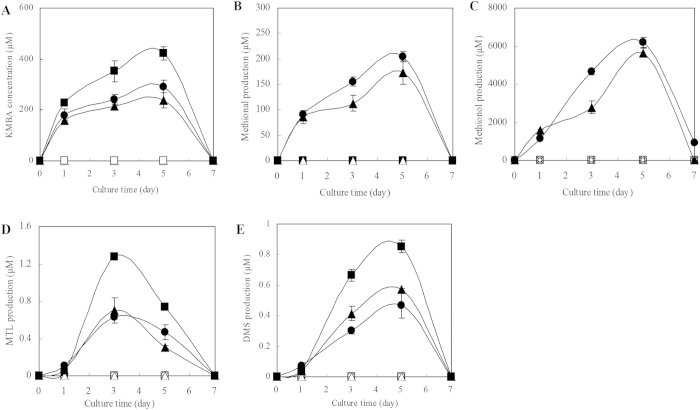
Effect of *HUWE1* overexpression on the production of (**A**) KMBA (**B**) Methional, (**C**) Methionol, (**D**) MTL, (**E**) DMS for *C. rosea*. ○,● *C. rosea* was inoculated without or with 5 g/L Met addition; △, ▲ *C. rosea* strain UH1 overexpressing *HUWE1* with HECT sequence deleted was inoculated without or with Met addition; □, ■ *C. rosea* strain U1 with *HUWE1* overexpression plasmid pCAMBIA1302-HUWE1 was inoculated without or with Met addition.

The effects of *HUWE1* overexpression on the biosynthesis of metabolites at metabolic and transcriptional levels were analyzed dynamically. After parent compound Met was added into the fermentation media, the production of KMBA (α-keto-methylthiobutyric acid), methional, methionol, MTL and DMS was observed for the wild type strain and strain UH1 overexpressing *HUWE1* with HECT domain sequence deleted, and both strains were with similar metabolite profiles. However, Met addition in fermentation medium made *HUWE1* overexpression strain U1 produce trace amount of methional and methionol of ehrlich pathway, but much more KMBA of intermediate, MTL and DMS of demethiolation pathway (Figs 3A–E and S4A–E). These results suggested that HUWE1 with HECT domain mediate the regulation of ehrlich and demethiolation pathways.

**Figure 4:**
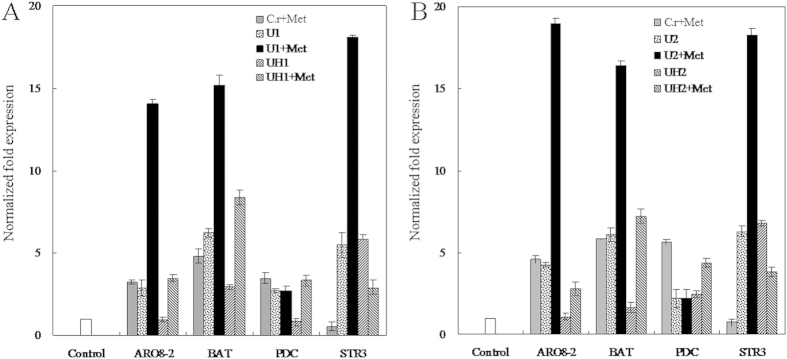
Transcriptional analysis of synthase genes in *HUWE1* overexpression strains U1 and U2, UH1 and UH2 with HECT domain sequence deleted. Normalized fold expression values for aminotransferase genes *ARO8-2* and *BAT*, decarboxylase gene *PDC* and demethiolase gene *STR3* in engineered strains U1, U2, UH1 and UH2 were relative to those in the wild type strain grown in the medium without Met addition. The error type was standard deviation.

Correspondingly, Met addition upregulated the expression of ehrlich pathway genes *ARO8-2*, *BAT*, *PDC* and downregulated the expression of demethiolation pathway gene *STR3* for the wild type strain and strain UH1 overexpressing *HUWE1* with HECT domain sequence deleted. Besides the upregulation expression of ehrlich pathway genes *ARO8-2* and *BAT*, Met addition made *HUWE1* overexpression strain U1 significantly upregulate the expression of demethiolation pathway gene *STR3*, but did not impact the expression of ehrlich pathway gene *PDC* (Fig. 4A,B). Similar metabolite and gene expression profiles were observed from another engineered strain U2 (Figs 4A,B and S4A–E). The transcriptional expression of synthase genes corresponded to metabolites production^15^,^25^. *HUWE1* overexpression accompanied with Met addition led to unchanged expression of decarboxylase gene *PDC* and upregulated expression of demethiolase *STR3*, which may account for that *HUWE1* overexpression strains accumulated more KMBA, MTL and DMS, but produced trace amount of methional and methionol. Strains UH1 and UH2 overexpressing *HUWE1* with HECT domain sequence deleted lost this capacity, indicating that HECT may play a direct role in catalysis during ubiquitination. Overexpressing *HUWE1* enhanced demetiolation pathway metabolites but inhibited ehrlich pathway metabolites, suggesting that HUWE1 was a key factor mediated the regulation of ehrlich and demethiolation pathways.

### Heterologous expression of *HUWE1* impacts the biosynthesis of pathway metabolites in *S. cerevisiae*

*S. cerevisiae* as a eukaryotic model and cell factory has been adopted to produce valuable alcohols and food aromatic thiol compounds via ehrlich and demethiolation pathways^3^,^25^. Regulators are of importance for metabolic engineering of yeast strains to increase metabolites production and tolerance, and control the quality of fermented foods^26^,^27^,^28^,^29^. Since the catalysis mechanism of HECT domain in HUWE1 was high conserved during ubiquitination, we tested whether heterologous expression of *HUWE1* with HECT domain sequence in *S. cerevisiae* could achieve the same effect under oenological conditions.

**Figure 5:**
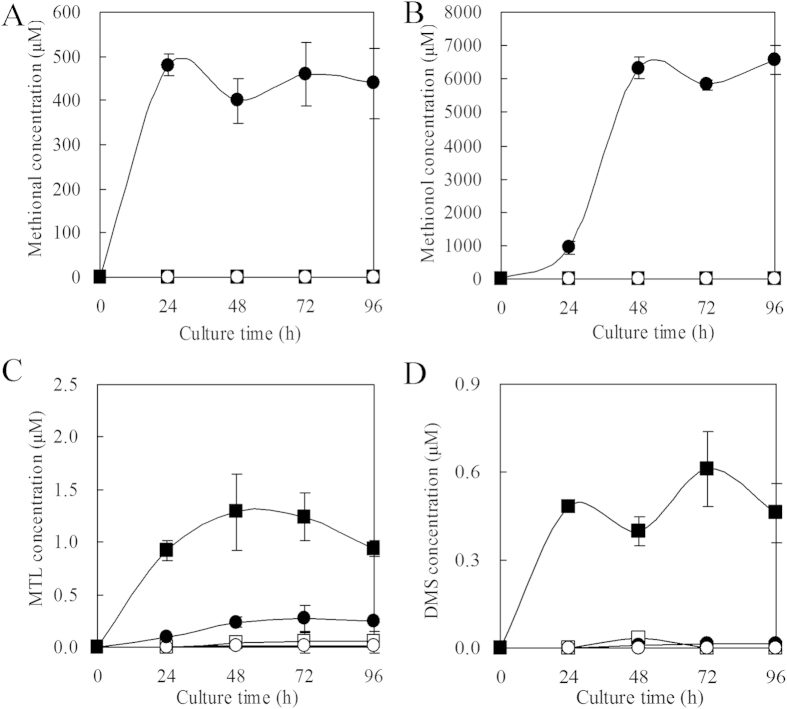
Effect of *HUWE1* overexpression on the production of (**A**) Methional, (**B**) Methionol, (**C**) MTL, (**D**) DMS in *S. cerevisiae*. ○ Control, *S. cerevisiae* with plasmid pYES2 was innoculated with 5 g/L Met; ● *S. cerevisiae* with plasmid pYES2 was inoculated with 5 g/L Met and 20 g/L galactose; □, *S. cerevisiae* with *HUWE1* overexpression plasmid was inoculated with 5 g/L Met; ■, *S. cerevisiae* with *HUWE1* overexpression plasmid was inoculated with 5 g/L Met and 20 g/L galactose.

**Figure 6:**
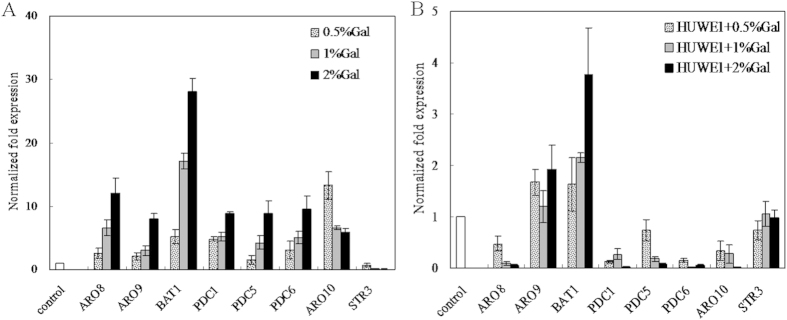
Transcriptional analysis of synthase genes in (**A**) plasmid control strain, (**B**) *HUWE1* overexpression strain of *S. cerevisiae*. The controls for A and B were engineered strains grown in SC-U medium without galactose (Gal) addition, respectively. Normalized fold expression values for aminotransferase genes *ARO8*, *ARO9*, *BAT1*, decarboxylase genes *PDC1*, *PDC5*, *PDC6*, *ARO10* and demethiolase gene *STR3* were relative to those of the controls. The error type was standard deviation.

The addition of galactose into fermentation medium led to the production of methionol and MTL for the plasmid control strain (containing pYES2) except KMBA which was not detected in Hebert’s studies^14^,^15^. As compared to that of the plasmid control strain, *HUWE1* overexpressing strain (containing pYES2-HUWE1) produced more MTL and its derivative DMS, but trance amount of methional and methionol of ehrlich pathway during whole fermentation process (Fig. 5A–D). Transcriptional analysis of synthase genes indicated that the production of methionol and MTL was accompanied by upregulation of the expression of aminotransferase genes *ARO8*, *ARO9*, *BAT1*, and pyruvate decarboxylase genes *PDC1*, *PDC5*, *PDC6*, *ARO10*, downregulation of the expression of demethiolase gene *STR3* for plasmid control strain (Fig. 6A). The addition of galactose with different concentrations into SC-U media triggered upregulation of *HUWE1* expression (Fig. S5). While overexpressing *HUWE1* led to downregulation of the expression of aminotransferase gene *ARO8* and pyruvate decarboxylase genes *PDC1*, *PDC5*, *PDC6*, *ARO10*, and has little effect on the expression of aminotransferase gene *ARO9* and demethiolase gene *STR3* (Fig. 6B). Relative to the plasmid control, overexpressing *HUWE1* significantly suppressed the expression of pyruvate decarboxylase genes of ehrlich pathway and alleviate the inhibition effect on demethiolation pathway gene. These data indicates that overexpression of *HUWE1* regulated ehrlich and demethiolation pathway metabolites.

## Discussion

**Figure 7:**
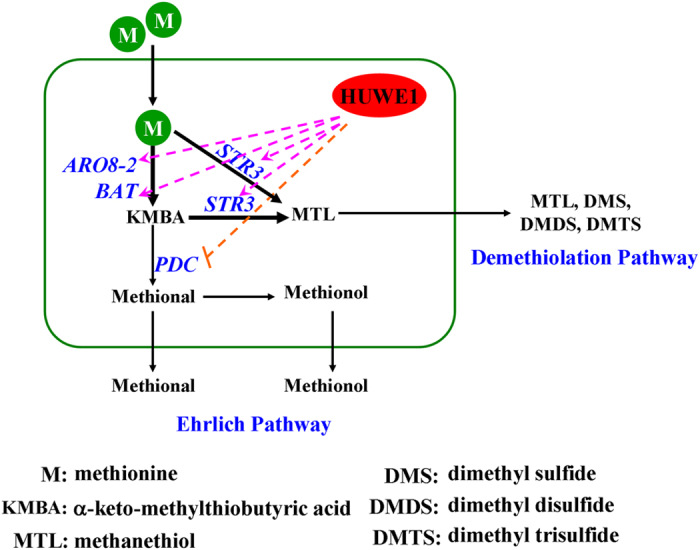
The overview of HUWE1 mediating the regulation of ehrlich and demethiolation pathways. Overexpression of *HUWE1* increased the expression of ehrlich pathway genes *ARO8-2* (*ARO9* in *S. cerevisiae*), *BAT* (*BAT1* in *S. cerevisiae*) and demethiolation pathway gene *STR3*, but suppressed the expression of ehrlich pathway gene *PDC* (*PDC1*, *PDC5*, *PDC6*, *ARO10* in *S. cerevisiae*). The corresponding metabolic products changed accordingly. This indicated that HUWE1 regulated the ehrlich and demethiolation pathways in eukaryotic cell.

In this study, one regulator E3 ubiquitin-protein ligase HUWE1 was screened from forward library of SSH and is inferred to play positive role during the biosynthesis of methionol and MTL. In *Clonostachys rosea* and *S. cerevisiae*, overexpressing *HUWE1* significantly increased the biosynthesis of MTL and its derivatives in demethiolation pathway, while suppressed the biosynthesis of methional and methionol in ehrlich pathway (Fig. 7). These results supported the directional regulation of ehrlich and demethiolation pathways.

Ehrlich pathway has been known for its ability to catabolize amino acid into valuable alcohols, demethiolation pathway as competing branch converted sulfur-containing amino acid into flavor thiols, thus the two pathways controlled the production of fuel and valuable flavor alcohols, the quality and uniqueness of many foodstuffs^1^,^2^,^3^,^4^,^5^. Regulators play key roles in metabolites production by controlling the expression of synthase genes. Modifying the behavior of regulators will create new phenotypes important for technological applications, due to that tuning the expression of regulator will reprogram the transcription of pathway genes^30^,^31^,^32^,^33^,^34^. This strategy has been successfully applied to optimization of several industrial phenotypes such as tolerance, metabolite over production, and so on^31^,^32^,^34^,^35^,^36^.

Taken the conversion of Met into methionol and MTL for example, pathway metabolite is complex and under multigenic control, engineering regulators would be a better choice for enhancing or controlling the conversion of Met into methionol and MTL^17^. One positive regulator ARO80 has been identified thus far for increasing ehrlich pathway metabolites^18^,^19^. In this study, a regulator gene *HUWE1* was selected, whose overexpression strengthen the expression of ehrlich pathway genes *ARO8-2* and *BAT* encoding aminotransferase, demethiolation pathway gene *STR3* encoding demethiolase but had no effect on the expression of ehrlich pathway gene *PDC* encoding α-ketoacid decarboxylase after Met was added into the fermentation medium. The transcriptional expression pattern of synthase genes corresponded to the production of pathway metabolites. This suggested that HUWE1 may control the biosynthesis of pathway metabolites at transcriptional level. Cellular homeostasis depends on a balance between anabolism and catabolism, which can be regulated by the ubiquitin-proteasome system (UPS). HUWE1, a key constituent of UPS, had a strong link with transcriptional control, cell cycle, lipid metabolism and so on, which was found to be involved in regulation of amino acid metabolism in this study^12^,^37^. Thus, HUWE1 is a potential metabolic switch for regulating cell metabolism.

A grand challenge in synthetic biology is to move the design of biomolecular circuits from purely genetic constructs toward systems that integrate different levels of cellular complexity, including regulatory networks and metabolic pathways^17^,^38^. The expression of synthase genes is often controlled at the transcriptional level by transcription factors that can sense either the end product or an intermediate metabolite, giving rise to different regulatory architectures^15^,^17^. E3 ubiquitin-protein ligase as a key constitution of ubiquitination system controlled the degradation of transcription factors and was located in upstream branch of regulatory architectures. Engineering it can achieve large-scale regulation of metabolites at cell level. Therefore, HUWE1 with HECT as regulatory architecture and motif discovered in this study has a great accomplishment in pathway engineering to improve cellular productivity and yield by exploiting dynamic pathway regulation and metabolic control.

## Methods

### Microorganism and culture condition

*Clonostachys rosea* tang 19 (Genbank accession number: KT007105) was grown on potato-agar-dextrose slants and produced methional, methionol, MTL and its derivatives from Met by inoculating it in fermentation medium (Met, 5 g/L; sucrose, 35 g/L; peptone, 2.5 g/L; yeast extract, 2.5 g/L; KH_2_PO_4_·H_2_O, 1 g/L; MgSO_4_·7H_2_O 0.5 g/L; vitamin B1, 0.05 g/L). *Escherichia coli* were grown in Luria-Bertani (LB) media containing 1% (v/w) tryptone, 0.5% yeast extract and 1.5% agar, and transformed strains were grown in LB medium supplemented with the appropriate antibiotic (s). *Agrobacterium tumefaciens* was grown and propagated in LB medium at 28 °C in the presence of appropriate marker selection supplements^39^. Commercial *S. cerevisiae* strain INVSc1 (Invitrogen) was used as a host strain for expressing *HUWE1* gene cassette. Yeast strains were cultivated at 30 °C in either a rich medium, YPD (Yeast Extract Peptone Dextrose Medium, containing 1% (w/v) yeast extract, 2% peptone, and 2% glucose), or a synthetic minimal defined medium, SC (synthetic complete medium, containing 2% glucose or raffinose, 0.67% yeast nitrogen base without amino acids (Biosharp)). Uracil (SC-U) was omitted to make selective plates for growing pYES2 transformants.

### Construction of suppression subtractive hybridization library

Total RNA from *C. rosea* was extracted by using E.Z.N.A.™ Fungal RNA Kit (Omega, USA), and RNA quality was confirmed by visualisation of ethidium bromide-stained bands following agarose gel electrophoresis and quantified by Biotek Synerg Spectrophotometer (OD260/OD280). Total RNAs extracted from *C. rosea* inoculated in fermentation medium were used as the tester. The deriver RNA was from *C. rosea* inoculated in fermentation medium without Met addition. Subsequently, the driver or tester RNAs were reversely transcripted to cDNA by SMARTer^TM^ PCR cDNA synthesis kit (Clontech, USA). 5′ PCR Primer II A from the kit was used for PCR amplification to enrich differentially expressed genes (Table S1). The optimal PCR cycles for the driver and tester were determined after dynamical detection of the PCR products by agarose gel electrophoresis (Fig. S1A). SSH was carried out using the PCR-selected cDNA Subtraction Kit (Clontech, USA) and then the cDNAs from tester and driver were digested by *Rsa*I (Fig. S1B). After two-round of SSH (Fig. S1C,D), the resulting PCR products were purified and inserted into pGEM-T vector (Promega, USA), then the product of a ligation reaction was transformed into *E. coli* DH5α. Recombinant colonies were selected and the length of inserted segments in the library was determined by PCR using nested primers (Table S1.).

### Sequencing of subtracted libraries

From the two subtracted cDNA libraries, 2405 recombinant bacterial clones were picked by blue–white selection on plates. These white colonies were grown and then 0.5 μL cultures were used as template for amplifying the exogenous segment by using the nested PCR-primers 1F and 2R (Table S1). PCR products were checked on 2% agarose gel and the remainder was used for screening the differentially expressed genes.

Screened by cDNA array dot blotting, 500 clones with cDNA inserted were selected randomly. The cDNA array dot blotting was conducted using forward- and reverse-subtracted cDNA probes prepared with the DIG High Prime DNA Labeling and Detection Starter Kit II (Roche Molecular Biochemicals, Germany). The hybridized signal intensity was analyzed with ChemiDoc Image Lab software (BioRad, USA) and spots that displayed at least threefold higher intensity with the forward subtracted probes than the reverse subtracted probes were scored as positives from forward-subtracted cDNA library. Using the same method, spots that displayed at least threefold higher intensity with the reverse subtracted probes than the forward subtracted probes were scored as positives from forward-subtracted cDNA library (Fig. S2A–D). And 100 clones with strongest hybridization signals were sequenced from forward- and reverse-SSH libraries, respectively.

### Relative quantification of mRNAs expression by real-time PCR

Four expressed sequence tags from the forward and the reverse libraries were selected for further analysis of their expression at transcriptional level by using quantitative real-time PCR, respectively. RNA was isolated by using the MasterPure Fungal RNA Purification Kit (Biozym Scientific GmbH). Complementary DNA (cDNA) was synthesized using a PrimeScriptTM 1^st^ Strand cDNA Synthesis Kit (TaKaRa Biotechnology (Dalian) Co., Ltd) with 0.5 μg of total RNA as the template. 18S rDNA served as standard gene for normalization of gene expression. Quantitative real-time PCR was performed on a StepOne Real-Time PCR system in duplicates for at least three independent experiments and the used primers were described in Table S1. SYBR dye (TaKaRa Biotechnology (Dalian) Co., Ltd) was used to visualize gene amplification. Relative quantification of the target gene expression was evaluated using the comparative cycle threshold (*C*_*T*_) method.

### Overexpression of *HUWE1*

A nested PCR-based strategy for genome walking named self-formed adaptor PCR was adopted to obtain the full-length of *HUWE1*^40^. Three primers including HUWE1-P5SP1, HUWE1-P5SP2, and HUWE1-P5SP3 (Table S1) were designed, which were located upstream of the cDNA sequence of *HUWE1*. The amplified PCR products were cloned into pGEM-T easy vector and then sequenced. After the whole cDNA was obtained, BLASTX program at the National Center for Biotechnology Information (NCBI) was used for functional inference. ORF Finder program was adopted to predict the ORF (Open Reading Frame) of *HUWE1* (Genebank accession number KT762629) and the ORF encoded a 988 amino acid polypeptide which showed 73% identity to its homologue in *Fusarium oxysporum* f. sp. EXM 30317.1.

The ORF corresponding to the entire coding region of *HUWE1* was flanked with *Xba*I and *Bst*EII sites by using PCR primers HUWE1*-*5F-XbaI and HUWE1-3R-BstEII (Table S1). After double digestion with *Xba*I and *Bst*EII, the PCR products were ligated into the linearized vector pCAMBIA1302 containing trpC promoter and terminator to create the expression vector pCAMBIA-HUWE1. pCAMBIA-HUWE1 was transformed into *C. rosea* with the help of *A. tumefaciens* strain AGL-1^39^. Transformants survived on the medium containing 50 mg/L hygromycin were identified by PCR with the primers of HPT-F2 and HPT-3 targeting hydromycin resistance gene, and then *HUWE1* overexpression at transcriptional levels were analyzed by quantitative real-time PCR. The selected HUWE1-overexpressed strains were named U1 and U2.

*HUWE1* with HECT sequence deleted was amplified by using PCR primers HUWE1*-*5F-XbaI and HUWE1-3BstEII (Table S1), and then inserted into fungal expression vector pCAMBIA1302 containing trpC promoter and terminator after double digestion with *Xba*I and *Bst*EII. The expression vector was transformed into *C. rosea* with the help of *A. tumefaciens* strain AGL-1. Overexpression of *HUWE1* with HECT sequence deleted was analyzed at transcriptional levels and the targeted strains were named UH1 and UH2.

### The effect of *HUWE1* overexpression on the biosynthesis of methionol and MTL in *C. rosea*

*HUWE1* overexpression strains U1 and U2, HECT deleted strains UH1 and UH2, and wild type strain Tang 19 were inoculated into the fermentation media. After the cultures were incubated for 0, 1, 3, 5 and 7 days, KMBA, methional, methionol, MTL and its derivatives were analyzed dynamically. Intracellular KMBA was extracted and detected following the previously mentioned method^15^. Fungal mycelia cultured in fermentation medium with 5 g/L Met were collected by filtration, washed with 20 mL of cold ultrapure water, and then filtered again. Mycelial pellets were stored at —80 °C. The pellets were resuspended with 1 mL of 1% (v/v) formic acid, incubated for 10 min at 95 °C and centrifuged for 30 min at 4 °C. The supernatant was lyophilized and stored at −80 °C. Before injection, samples were resuspended in water containing 0.1% formic acid and stored at 4 °C. Chromatographic separation was performed on a Reprosil-Pur Basic C18 column (4.6 mm × 250 mm × 5 μm) from Dr. Maisch GmbH (Germany) using a liquid chromatography 20AD system (Shimadzu Corporation, Japan). The optimized mobile phase was 0.1% formic acid water solution. The column oven temperature was set to 30 °C, and the flow rate was 1 mL/min. The detection wavelength was 197 nm. The retention time for KMBA was 12.5 min. Methional, methionol, MTL and its derivatives were extracted and detected as follows. A 5-mL volume of fermentation culture was added into a 15-mL vial, the vial was tightly capped with a silicon septum and pre-equilibrated at 40 °C for 5 min, and then solid-phase microextraction (SPME) device was inserted into the headspace of vial to extracte MTL and its derivatives for 20 min at 40 °C. MTL and its derivatives were analyzed by gas chromatography (GC-2010 with a flame ionization detector (FID) from Shimadzu Technologies Inc., Tokyo, Japan) with an oven temperature program. First, the temperature was maintained at 35 °C for 3 min. Subsequently, the temperature reached 100 °C, with an increment of 8 °C/min. Then, the temperature was raised to 220 °C, with an increment of 10 °C for 1 min. Additionally, ehrlich pathway genes including *ARO8-2* (Genebank accession number KT157523) and *BAT* (KT157521) encoding aminotransferase, *PDC* (KT762630) encoding a-keto acid decarboxylase and demethiolation pathway genes *STR3* (KT157524) encoding demethiolase were analyzed at transcriptional level by quantitative real-time PCR.

### Effect of *HUWE1* overexpession on the biosynthesis of methionol and MTL in *S. cerevisiae*

The isolation and manipulation of DNA from *C. rosea* were performed using E.Z.N.A Fungal DNA kit (Omega) according to the manufacturer’s protocol. Primer pairs YHUWE1-5HK and YHUWE1-3XH were designed to amplify the complete ORF of *HUWE1* from chromosomal DNA of *C. rosea* (Table S1). *Kpn*I (in the forward primer) and *Xba*I (in the reverse primer) restriction sites were introduced to ensure that *HUWE1* was correctly inserted into yeast expression plasmid pYES2. Engineered *S. cerevisiae* strains containing control plasmid pYES2 or *HUWE1* overexpression plasmid pYES2-HUWE1 were inoculated into SC-U medium containing 2% (w/v) glucose, respectively. After overnight culture, cells were collected and the pellets were resuspended into SC-U medium (Optical Density of 0.4 at 600 nm) containing 0.5% Met, and then 2.0% galactose were added into the SC-U medium for inducing the expression of *HUWE1*. Methional, methionol, MTL and its derivatives were extracted and dynamically analyzed following the method described above. Additionally, different concentrations of galactose (0, 0.5, 1.0, 2.0% (w/v)) were added into the SC-U media for culturing *HUWE1* overexpression strain and plasmid control strain. After 3 days of cultivation, total RNA were extracted and then synthase genes including *ARO8*, *ARO9* and *BAT1* encoding aminotransferase, *PDC1*, *PDC5*, *PDC6* and *ARO10* encoding pyruvate decarboxylase and *STR3* encoding demethiolase were analyzed by real-time PCR followed the method described above (Table S1).

## Additional Information

**How to cite this article**: Zhang, Q. *et al.* Regulating ehrlich and demethiolation pathways for alcohols production by the expression of ubiquitin-protein ligase gene HUWE1. *Sci. Rep.* **6**, 20828; doi: 10.1038/srep20828 (2016).

## Supplementary Material

Supplementary Information

## Figures and Tables

**Table 1:** Differentially expressed genes selected from forward SSH cDNA library.

cDNA clones	Annotation	Functional group
FL670, 737, 762, 843, 871	heat shock protein 30	Cellular organization
FL693	SIT4-associating protrein	
FL733, 816	collagen triple helix repeat-containing protein	
FL666	pyruvate decarboxylase	Metabolism
FL671	aromatic amino acid aminotransferase	
FL731	aminotransferase class I and II	
FL819	3-isopropylmalate dehydrogenase	
FL870	short-chain dehydrogenase/reductase	
FL675, 688, 745, 768, 861, 878	metallo-beta-lactamase domain protein	
FL719, 806, 839	YLR154W-A-like protein	
FL844	sulfide quinone-reductase	
FL735, 803, 822, 826	cysteine dioxygenase	
FL 746	cytochrome c oxidase subunit Va	
FL747	vacuolar protein sorting-associated protein	
FL776	long-chain fatty acid-CoA ligase	
FL849	Ubiquinol-cytochrome C reductase, UQCRX/QCR9-like protein	
FL857	glycoside hydrolase, family 5	
FL864	NADH dehydrogenase subunit 4 (mitochondrion)	
FL346	E3 ubiquitin-protein ligase HUWE1	Protein fate
FL707	Ubiquitin 3 binding protein But2	
FL643	F-box domain containing protein	Transcription
FL820	PWWP (Pro-Trp-Trp-Pro) domain-containing protein	
FL639, 703, 757, 736, 829, 865	Tar1p (transcript antisense to ribosomal RNA protein)	
FL628, 818	riboflavin transporter MCH5	Transport facilitator
FL655	MFS monocarboxylate transporter	
FL685, 814	major facilitator superfamily transporter	
FL730	RND transporter	
FL754,778, 869, 876	related to monocarboxylate transporter 4	
FL808, 828	protein cft1	

The length of SSH cDNA clones was determined by DNA sequencing with an automated 3730 DNA sequencing system. The other 36 differentially expressed genes were annotated as hypothetical protein or had no match with functional known protein.

**Table 2:** Differentially expressed genes selected from reverse SSH cDNA library.

cDNA clones	Annotation	Functional group
RL171, 250	mannose-binding lectin	Cell defense
RL138, 148, 158, 203, 206, 260, 383	polyketide synthase	Cellular organization
RL162	related to hsp70 protein	
RL167, 182, 186, 192, 252, 255, 264, 266, 273, 340, 359	amino-acid permease inda1	
RL181	N amino acid transport system protein	
RL275	leucine-rich repeat protein	
RL312	WD domain-containing protein	
RL341	Hypothetical protein SETTUDRAFT-166173	
RL381	hypothetical protein NECHADRAFT-36802	
RL 142	anaerobic dehydrogenase	Metabolism
RL191	serine-type peptidase-like protein	
RL202	NAD(P)-binding Rossmann-fold containing protein	
RL213, 238, 323	aminotransferase class I and II	
RL224	quinone oxidoreductase 2	
RL272, 350, 377	5-aminolevulinate synthase	
RL279	serine peptidase	
RL309	glutathione S-transferase	
RL336	nitrite reductase NiiA	
RL 347	cytochrome P450	
RL400	glycoside hydrolase family 16 protein	
RL406	acetyl-CoA carboxylase	
RL263	ubiquitin conjugating E2	Protein fate
RL146	Elongation factor 2	Protein synthesis
RL319, 345	ribonuclease t2	
RL153	tripeptidyl-peptidase 1 precursor	Signal transduction
RL253	serine/threonine protein kinase	
RL288	GPR1/FUN34/yaaH family protein	
RL 164	Hms1p	Transcription
RL404	transcription factor C6	
RL163	related to multidrug resistance protein fnx1	
RL166	purine nucleoside permease	
RL170	related to zinc transporter	
RL173	PiT family inorganic phosphate transporter	
RL190	glutamyl-tRNA (Gln) amidotransferase subunit A	
RL205	OPT family small oligopeptide transporter	
RL207	purine nucleoside permease	
RL208, 348	phosphatidylglycerol/phosphatidylinositol transfer protein	
RL211, 363	ATPase	
RL217	related to dopamine-responsive protein	
RL232	related to C4-dicarboxylate transport protein mae1	
RL322	ABC-2 type transporter	
RL330	NCS1 family nucleobase:cation symporter-1	
RL335	MFS general substrate transporter	
RL337	sulfate permease	
RL385	cobalamin synthesis protein	

The length of SSH cDNA clones was determined by DNA sequencing with an automated 3730 DNA sequencing system. The other 23 differentially expressed genes were annotated as hypothetical protein or had no match with functional known protein.
