# Supplementary material for: Regulating ehrlich and demethiolation pathways for alcohols production by the expression of ubiquitin-protein ligase gene HUWE1
Source: Sci Rep. 2016 Feb 10;6:20828. doi: 10.1038/srep20828 (PMC4748413; doi:10.1038/srep20828)
Supplement: Supplementary Information [file srep20828-s1.pdf]

Regulating ehrlich and demethiolation pathways for alcohols production by the expression of ubiquitin-protein ligase gene *HUWE1*

Quan Zhang[1], Kai-Zhi Jia[1], Yang-Hua Xu, Rui-Sang Liu, Hong-Mei Li & Ya-Jie Tang*

Key Laboratory of Fermentation Engineering (Ministry of Education),

Hubei University of Technology, Wuhan 430068 China

*Corresponding author. Tel. & Fax: +86-27-59750491. Email: yajietang@QQ.com

[1] With equal contribution to this work

Figure legends

Figure S1. The enrichment of differentially expressed genes putatively involved in the production of methionol and MTL. (A) The synthesis of SMARTer cDNA (9, 12, 15, 18, 21 indicates PCR cycle number); (B) Agarose gel eleetrophoresis of ds cDNA before (lane 1, 3) and after (lane 2, 4) *Rsa*I digestion (lane 1 and 2, control; lane 3 and 4, methionine addition); (C) and (D) Results of first and second round of suppressive subtractive hybridization array (lane1 and 3, PCR products of subtracted samples (with or without Met addition)); lane2 and 4, PCR products of unsubtracted samples (with or without Met addition))

Figure S2. Screening of differentially expressed genes putatively involved in the production of methionol and MTL. Equal amount of amplified cDNAs were arrayed onto two sets of membranes and hybridized with two different probes. (A, C): Blots hybridized with forward subtracted cDNA probe. (B, D): Blots hybridized with reverse subtracted cDNA probe.

Figure S3. Identification of *HUWE1* mRNA levels in *HUWE1* overexpression strains U1 and U2, UH1 and UH2 with HECT sequence deleted (C. r, *Clonostachys rosea* ; +M, methionine addition). Normalized fold expression values for *HUWE1* in engineered strains U1, U2, UH1 and UH2 were relative to that in the wild type strain of *Clonostachys rosea* grown in the medium without Met addition. The error type was standard deviation.

Figure S4. Effect of *HUWE1* overexpression on the production of (A) KMBA (B) Methional, (C) Methionol, (D) MTL, (E) DMS for strains U2 and UH2 (○ Control, *C. rosea* was grown in the medium without Met addition; ● *C. rosea* was grown in the medium with 5 g/L Met; △, ▲ *C. rosea* strain UH2 overexpressing *HUWE1* with HECT sequence deleted was grown in the medium without or with Met addition; □, ■ *C. rosea* strain U2 with *HUWE1* overexpression plasmid pCAMBIA1302-HUWE1 was grown in the medium without or with Met addition).

Figure S5. Transcriptional analysis of *HUWE1* in *S. cerevisiae*. Normalized fold expression values for *HUWE1* in *HUWE1* overexpression strain of *S. cerevisiae* were relative to that in the plasmid control strain grown in the SC-U medium without Met addition. The error type was standard deviation.

Table S1 Primers used for quantitative real-time PCR and cloning.

Gene	Primer name	Primer sequence (5’→3’)
18S rRNA	18S rRNA-F	CTTCGGGGCTCTCTTGGTGAT
	18S rRNA-R	TGCTGTTCCTTGGATGTAGT
nested PCR-primers for SSH	1F	TCGAGCGGCCGCCCGGGCAGGT
	2R	AGCGTGGTCGCGGCCGAGGT
FL346 (*HUWE1*)	FL346-5F	TCAATCAGCTCCCAAGAAGCAGTTAAGAC
	FL346-3R	CAGAGCTACAATAACGCCTCGATGGAAAG
FL671 (*ARO8-2*)	FL671-5F	GCATGGACTCCTTCAGCAAAGTC
	FL671-3R	CATTGCTCGTCAAGGAGTTTGAAG
FL666 (*PDC*)	FL666-5F	AGCTGAATCGCCCCTCTGAAATTGCGGAC
	FL666-3R	GACGACATCCACTACATAGTCTTCCTTC
FL819 (*IDH*)	FL819-5F	AAGGGTATTTATGAGCCGGTGCATGG
	FL819-3R	ATACTTTGACAACGGCCTGTTCCACG
RL186	RL186-5F	GTGCTATCCGACAGGTGTTCTGGC
	RL186-3R	CCGGCATACTTGCCGACAAGCACAAA
RL323	RL323-5F	AGCCTCGCAGCCGAAAGGATTGAGT
	RL323-3R	CCTTGGATACATCAACAAGCTCCTG
RL272	RL272-5F	ATACATTTGGCAAGGCCATGGGTTG
	RL272-3R	GCACTGAGCAAGTTATAGCCAGCTCG
RL138	RL138-5F	TAAGCGATATCTTGGAAGCCAACACCCC
	RL138-3R	AACTCAAGTCTCCGGGCGGAGCTCAA
HUWE1	HUWE1-P5SP1	ACGCAATATAACGGCAAGGTCGAC
	HUWE1-P5SP2	CATCTTCCTTCTCCCCGAGAAAGCCAATC
	HUWE1-P5SP3	CCATCGAGGCGTTATNNNNNNNNNGCAAAG
	HUWE1-5F-XbaI	CTAG*TCTAGA*ATGGAAGGAATTGAAGGAGACGAG
	HUWE1-3R-BstEII	TTG*GGT(A/T)ACC*TTAGAGGGGGAAAACAATGCACTTAC
	HUWE1-3BstEII	TTG*GGT(A/T)ACC*GGTGGGGTAAGAAGGCCGGCCGTGG
hpt	HPT-F2	GACGACACCGTCAGTGCGTC
	HPT-3	CTTTGCCCTCGGACGAGTGCTGG
BAT	BAT-3NR	CCAGGCTCAGCCCTCCTCTATGTTATTG
	BAT-5NR	CAGGTTCTGCTGGTGGCCCCTGGAGCTG
STR3	STR3-3NR	CCAACAATCGCCCGGAATGCCCACGAAG
	STR3-5NR	TCATTGGGTGACAGACCGCAGCCAGTTG
YBAT1	YBAT1-5F	GCCTACAGAACTCCTCAAAATACTATC
	YBAT1-3R	GAGGAACCAAGTGTTTATCTTGTTCGATC
YARO8	YARO8-5F	GTCAAGAGCTTTGCAATACGGGTTCAGTG
	YARO8-3R	GATTCCCAGGCATTTGTGTTACCTGCAG
YARO9	YARO9-5F	CTACCCATTGCACGTTCTTTCCAATATG
	YARO9-3R	GAAAGGATGACATCCCAGTTAGACTCCG
YPDC1	YPDC1-5F	CTCGTATCAAGGGTATGTCTTGTATCATC

	YPDC1-3R	CTTGAGCAGAGATGGATGGGACACCAACA
YPDC2	YPDC2-5F	TGCACAAGCTGTTTGGCACAGAATTCCTG
	YPDC2-3R	GTTTTGGGTAACTCTTCGTCTAAAACAG
YPDC5	YPDC5-5F	GTTACGCTCGTATCAAGGGTATGTCCTG
	YPDC5-3R	CAATTGCTTAGCTTGAGAAGAGATGGATG
YPDC6	YPDC6-5F	CGCATCAAGGGTTTATCTGTGCTGGTAAC
	YPDC6-3R	GAGGGGACACCAACAACATGCAGTACAC
YARO10	YARO10-5F	ACGTATGGCGTTGGTGAATTAAGCGCCTT
	YARO10-3R	CAAATGATGTAGGTTCCGATCACTAAAGT
YSTR3	YSTR3-5F	GTCTTACTTAACGGCACTGACAACCATAC
	YSTR3-3R	GAAGTGTCCACATGAACAGAGACTGCATG
Y18S rRNA	Y18S rRNA-5F	GAAACGGCTACCACATCCAAGGAAG
	Y18S rRNA-3R	GTTCCTCGTTAAGGTATTTACATTG
HUWE1		GGGGTACCAACACAATGTCTGAAGGAATTGAAG
	YHUWE1-5HK	GAGACGAGGCCAAC
	YHUWE1-3XH	GCTCTAGATTAGAGGGGGAAAACAATGC

The restriction enzyme sites are in italics, synthase genes derived from *Saccharomyces cerevisiae* are tagged with Y.

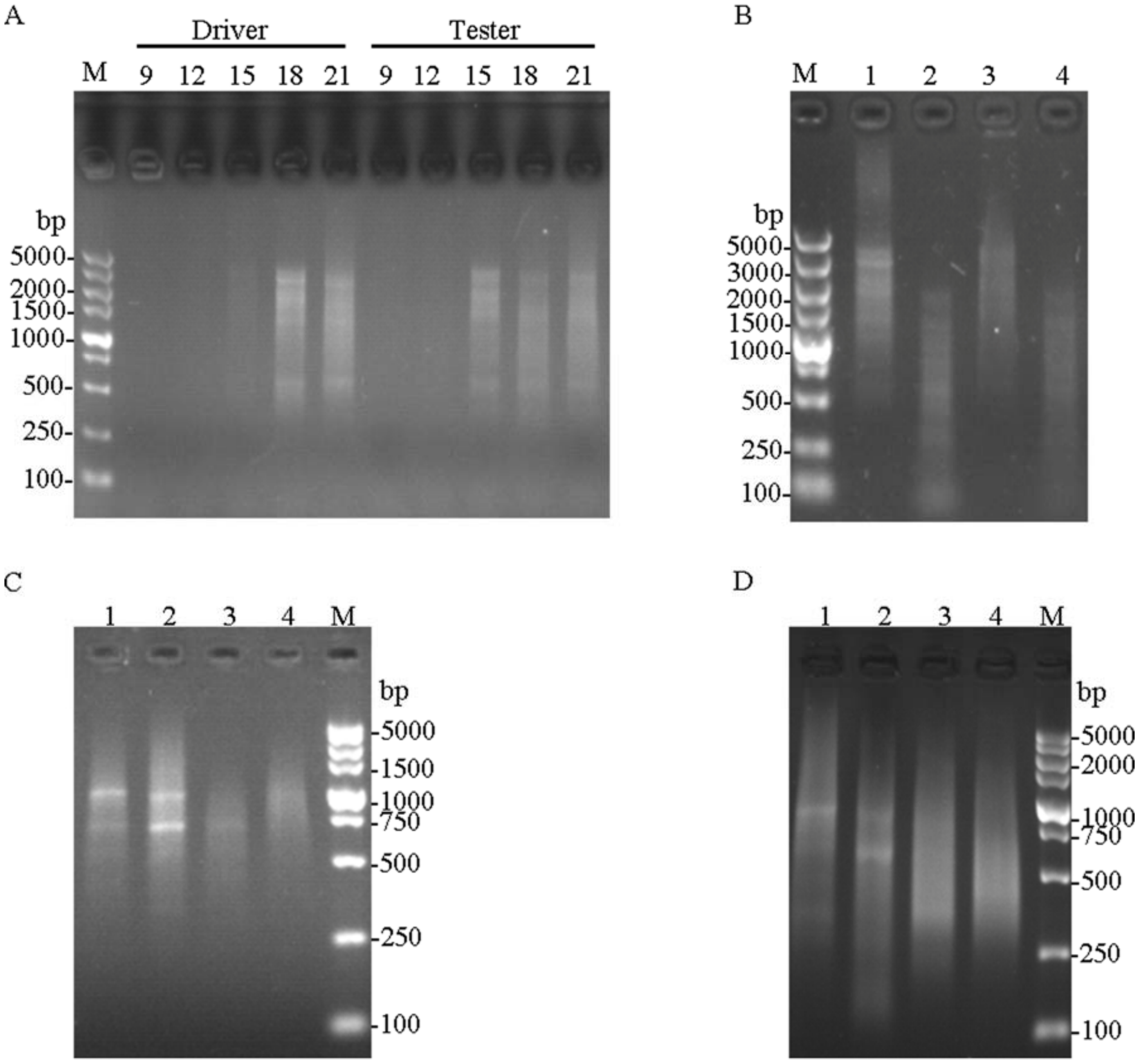

Figure S1. The enrichment of differentially expressed genes putatively involved in the production of methionol and MTL.

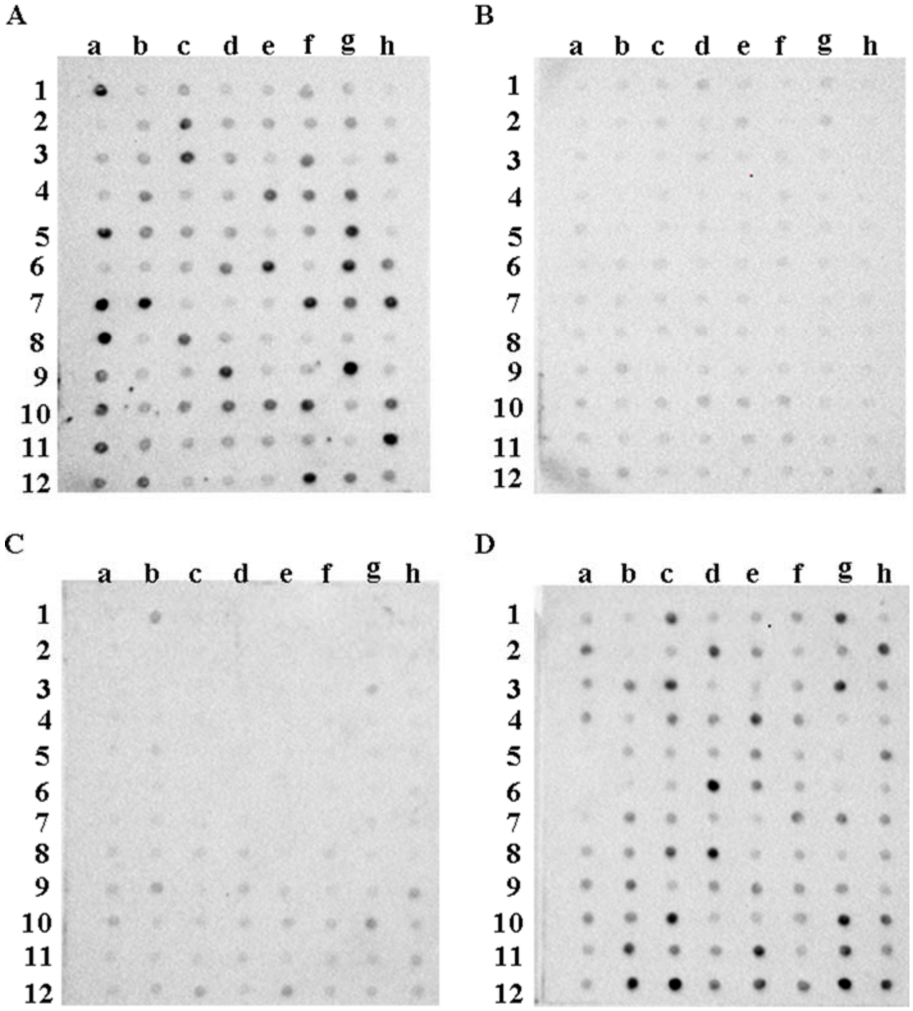

Figure S2. Screening of differentially expressed genes putatively involved in the production of methionol and MTL.

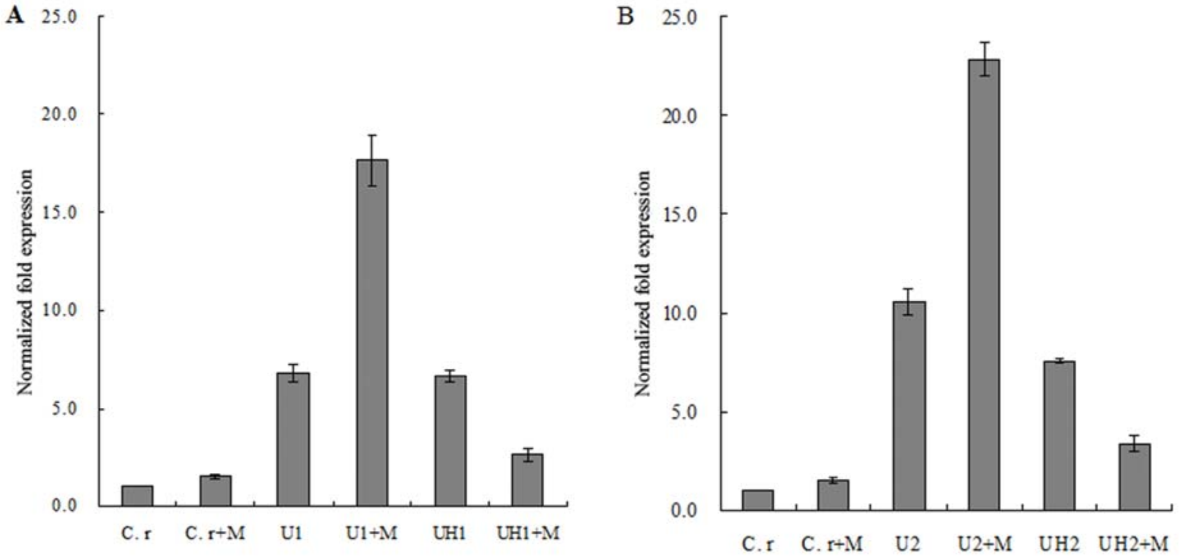

Figure S3. Identification of *HUWE1* mRNA levels in *HUWE1* overexpression strains U1 and U2, UH1 and UH2 with HECT sequence deleted.

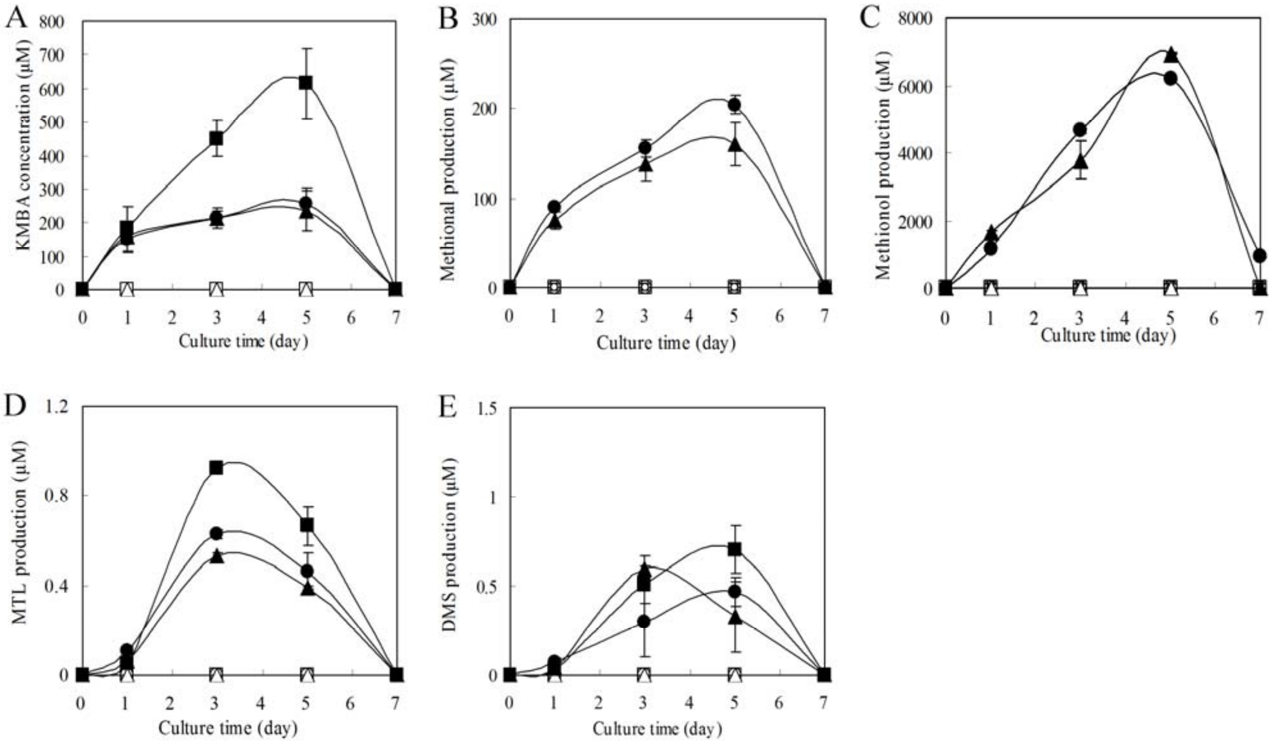

Figure S4. Effect of *HUWE1* overexpression on the production of (A) KMBA (B) Methional, (C) Methionol, (D) MTL, (E) DMS for strains U2 and UH2.

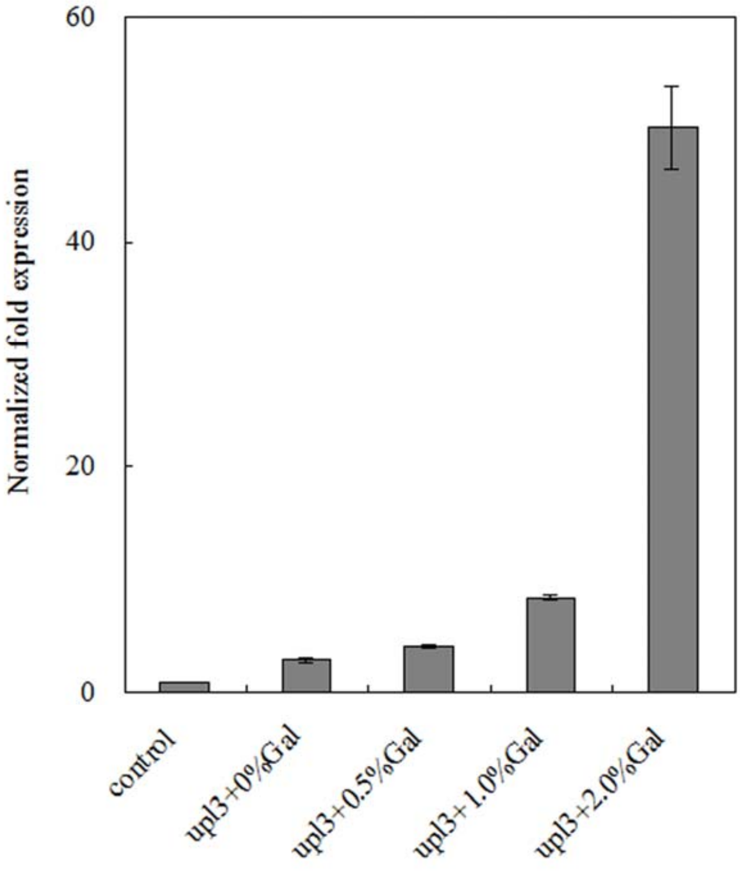

Figure S5. Transcriptional analysis of *HUWE1* in *S. cerevisiae*.
